# Sagittal alignment patterns in forward head posture: an EOS-based head-to-pelvis correlation analysis

**DOI:** 10.1186/s12891-026-10267-4

**Published:** 2026-08-05

**Authors:** Katterine N. Rios-Peralta, Ali Firouzabadi, Kathleen M. Curran, David B. MacManus, Lukas Schönnagel, Matthias Pumberger, Hendrik Schmidt

**Affiliations:** 1 School of Medicine, University College Dublin, Dublin, Ireland; 2 Julius Wolff Institute, Berlin Institute of Health at Charite´- Universitätsmedizin Berlin, Berlin, Germany; 3 UCD Centre for Biomedical Engineering, University College Dublin, Belfield, Dublin 4 Ireland; 4 School of Mechanical & Materials Engineering, University College Dublin, Belfield, Dublin 4 Ireland; 5 UCD Conway Institute of Biomolecular and Biomedical Research, University College Dublin, Belfield, Dublin 4 Ireland; 6 Center for Musculoskeletal Surgery, Charité - Universitätsmedizin Berlin, Berlin, Germany

**Keywords:** Forward head posture, Sagittal balance, Upper cervical spine, T1 slope, Global alignment, EOS imaging, Low back pain

## Abstract

**Study Design:**

Retrospective radiographic study.

**Objectives:**

Forward head posture (FHP) is commonly observed in individuals with prolonged screen exposure and may affect spinal alignment. Because the craniovertebral angle (CVA) alone does not fully capture how sagittal balance is distributed across the spine, this study evaluated head-to-pelvis sagittal alignment patterns in individuals with and without FHP, with particular attention to the upper cervical region.

**Methods:**

This study evaluated head-to-pelvis sagittal balance (SB) patterns in individuals with and without FHP using standing whole-body EOS radiographs. A total of 201 adults were referred for evaluation of low back pain (LBP) and classified into FHP and non-FHP groups using a predefined CVA threshold as a pragmatic stratification criterion. Sixteen SB parameters spanning the cranio-cervical, thoracic, lumbo-pelvic, and global regions were analysed using group comparisons, age-adjusted regression, and correlation analyses.

**Results:**

Individuals with FHP showed greater upper cervical extension and higher values of cervical sagittal vertical axis, T1 slope, thoracic kyphosis, and global sagittal vertical axis, while lumbar lordosis and spinopelvic parameters remained comparable between groups. In non-FHP individuals, subaxial cervical alignment (C2–C7) was associated with global alignment. However, these relationships were absent in the FHP group, with correlations shifting toward the upper cervical parameters (C0-C1, C0-C2) including associations with pelvic parameters, and the T1 slope, which showed stronger associations across cervical and global alignment.

**Conclusions:**

These findings suggest a redistribution of sagittal alignment relationships toward the upper cervical and cervico-thoracic regions in FHP, while lumbar structural alignment remains preserved, highlighting the limitations of CVA-centric frameworks for capturing whole-spine postural adaptations.

## Introduction

Musculoskeletal conditions represent one of the leading sources of economic burden and reduced productivity worldwide [1, 2]. Among postural deviations, forward head posture (FHP) is common, with high prevalence reported in young adults, including rates approaching 73% [3]. This postural condition is often linked to prolonged screen exposure [4] and suboptimal occupational ergonomics [5]. Alterations in head and neck positioning due to FHP significantly modify cervical alignment [6, 7]. This misalignment was associated with neck-related pain, disability, and degeneration, which may contribute to reduced physical function and poorer neck-related health status [8]. These conditions are estimated to affect 269 million individuals globally by 2050 [9].

Sagittal balance (SB) is the assessment of the spine from the lateral view, often using radiographic imaging. This approach is used in clinical practice and for mechanical interpretation in the standing position [10]. In this context, SB becomes an important evaluation in FHP, where the body’s center of mass is mechanically shifted forward, bringing the head forward relative to the trunk [11, 12]. This displacement is accompanied by gaze horizontalization control, which accommodates the head within the overall spinal balance. Biomechanically, the craniovertebral junction [13, 14], particularly the C0-C2 cervical, is responsible for this coordination to maintain horizontal gaze. This region is defined as the upper cervical area and plays a unique functional role due to its distinct anatomy and mechanics. The upper cervical region differs from the typical vertebral structure due to its larger range of motion [15, 16].

In FHP, the skull condyle allows an anteroposterior rotation towards the posterior part of the condyle [15]. This shift often leads to increased extension of C0-C2 lordosis, while subaxial cervical spine C2-C7 lordosis decreases, acting inversely [11, 17]. As most SB studies take C2-C7 alignment as a representation of the entire cervical spine, the upper cervical region C0-C2 remains overlooked [16, 18], despite its key role in gaze-related postural adaptation [19]. Such reciprocal behaviour between the upper and lower cervical spine suggests an alignment pattern that may extend beyond the cervical region, potentially involving thoracic and lumbo-pelvic alignment as part of a whole-spine sagittal alignment framework [20, 21].

Even though the interdependence of spinal alignment is a well-known feature in SB analysis, the role of C0-C2 and its relationship with the whole-spine alignment remains poorly understood [18]. In line with this concept of whole-spine involvement, associations between FHP and low back pain (LBP) have been reported previously [22]. This finding is of interest given the growing burden of LBP [23], and the importance of the lumbar region in spinal load redistribution. However, that analysis included only a FHP cohort without a non-FHP comparison group, did not incorporate upper cervical alignment parameters, and characterized cervical posture exclusively through the craniovertebral angle (CVA). Importantly, no universal CVA cutoff has been established to diagnose FHP [24]. This may reflect the fact that FHP represents a continuum of postural presentations, where CVA-based thresholds are more appropriately viewed as distinguishing lower-CVA and higher-CVA postural phenotypes rather than definitive diagnostic categories. Nevertheless, CVA serves as a useful descriptor of FHP classification but alone offers limited insight into the underlying mechanical organization. Therefore, we hypothesized that individuals with FHP would exhibit (i) global SB alterations compared with individuals without FHP and (ii) distinct inter-regional alignment relationships, particularly involving C0-C2 and cervico-thoracic SB parameters, potentially extending to lumbar lordosis and spinopelvic alignment. Using low-dose, standing biplanar EOS imaging, the present study addresses these limitations by providing an integrated radiographic analysis of head-to-pelvis alignment in individuals with and without FHP, with explicit inclusion of upper cervical, thoracic, lumbar, and spinopelvic SB parameters, alongside a comprehensive correlation landscape and an individualized analysis of “ideal” lumbar sagittal alignment.

## Methodology

### Study participants

We conducted a retrospective radiographic study of sagittal spinal alignment using standing lateral EOS images from participants referred for evaluation of LBP at a tertiary orthopaedic outpatient spine clinic. Ethical approval was obtained from the Charité–Universitätsmedizin Berlin Ethics Committee (EA4/011/10, EA1/162/13, EA2/021/22). All procedures were performed in accordance with the Declaration of Helsinki and relevant institutional guidelines and regulations. All participants provided informed consent prior to inclusion in the study. A total of 408 consecutive EOS radiographs were initially screened, of which 201 met the predefined inclusion and exclusion criteria and were retained for final analysis. All data were analysed retrospectively and in anonymized form.

**Fig. 1:**
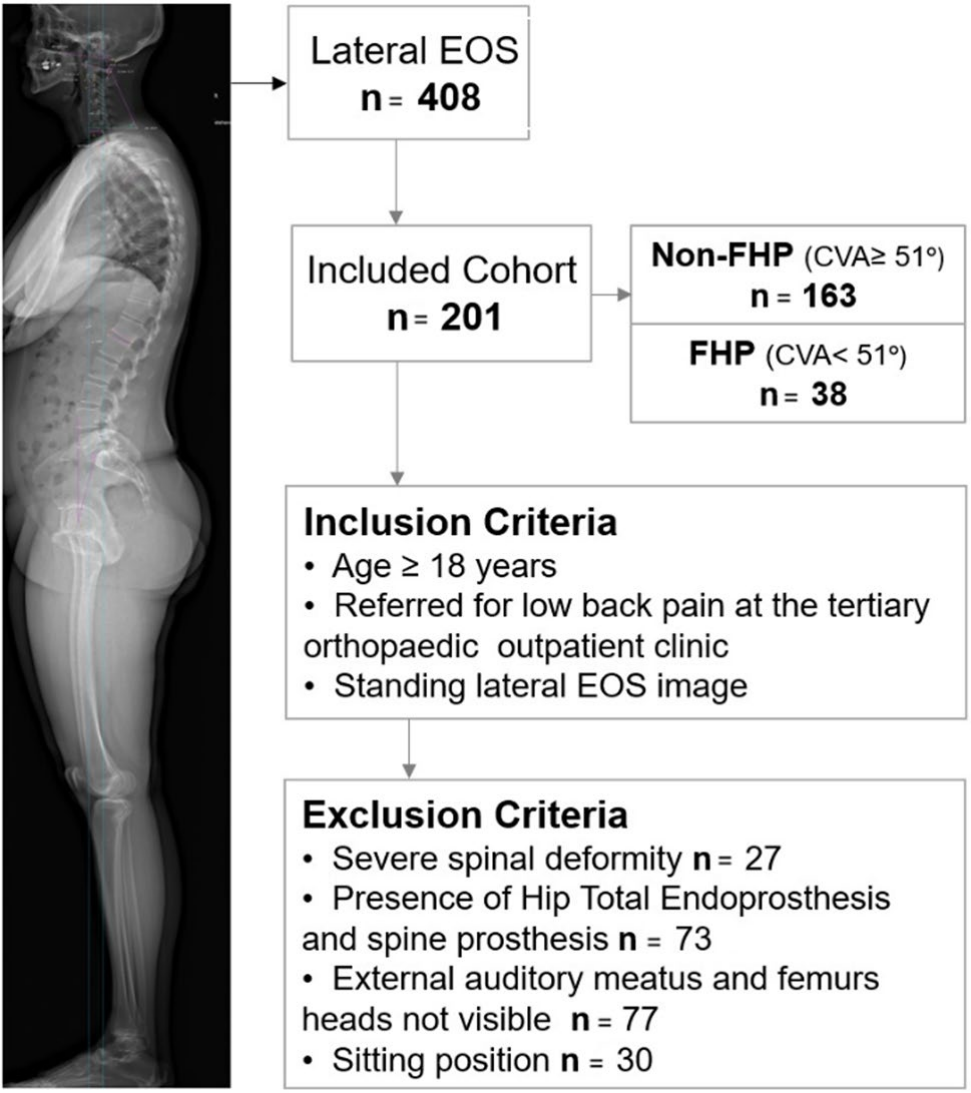
Study flowchart illustrating cohort selection, stratification, and exclusion criteria

Participants were eligible for inclusion if they were 18 years of age or older and had a standing lateral EOS radiograph of sufficient quality for sagittal alignment analysis. Exclusion criteria included: (i) severe spinal deformity; (ii) presence of hip or spinal prosthetic implants; (iii) absence of a clearly visible external auditory meatus or femoral heads required for CVA and spinopelvic measurements; (iv) non-standing image acquisition (e.g., sitting position); and (v) inadequate image quality precluding reliable radiographic assessment (Fig. 1).

Spinal deformities were not classified using Cobb-based criteria, as the study aimed to explore sagittal alignment patterns rather than define deformity entities. Instead, a landmark-based screening approach was applied. Radiographs were excluded when advanced degenerative changes or structural alterations (e.g., large or bridging osteophytes, marked vertebral shape distortion). This method prevented reliable identification of the anatomical landmarks required for SB measurements.

### Group classification (FHP vs. Non-FHP)

Participants were stratified into two groups based on the CVA, measured on standing lateral EOS radiographs: a FHP group (FHP; CVA < 51°) and a non-FHP group (CVA ≥ 51°). The CVA threshold of 51° was selected as an intermediate, pragmatic cutoff derived from commonly reported values in the literature. Some studies define FHP using a higher cutoff (e.g., CVA < 53°) [24], whereas others apply stricter criteria (e.g., CVA < 48°) [25] to indicate FHP. Given the absence of a universally accepted CVA cutoff, this threshold was adopted to enable cohort stratification for comparative analysis rather than to establish a diagnostic definition of FHP.

### Sagittal balance measurements

Sagittal alignment measurements were derived from standing lateral EOS radiographs using a landmark-based approach. All radiographic images were processed in their original DICOM format. Image visualization and manual landmark annotation were performed using custom-written scripts in Python (Python Software Foundation). Spatial calibration was performed on a per-image basis using pixel spacing information extracted from the DICOM metadata, allowing conversion of pixel-based coordinates into metric units (millimetres) for linear measurements.

**Fig. 2:**
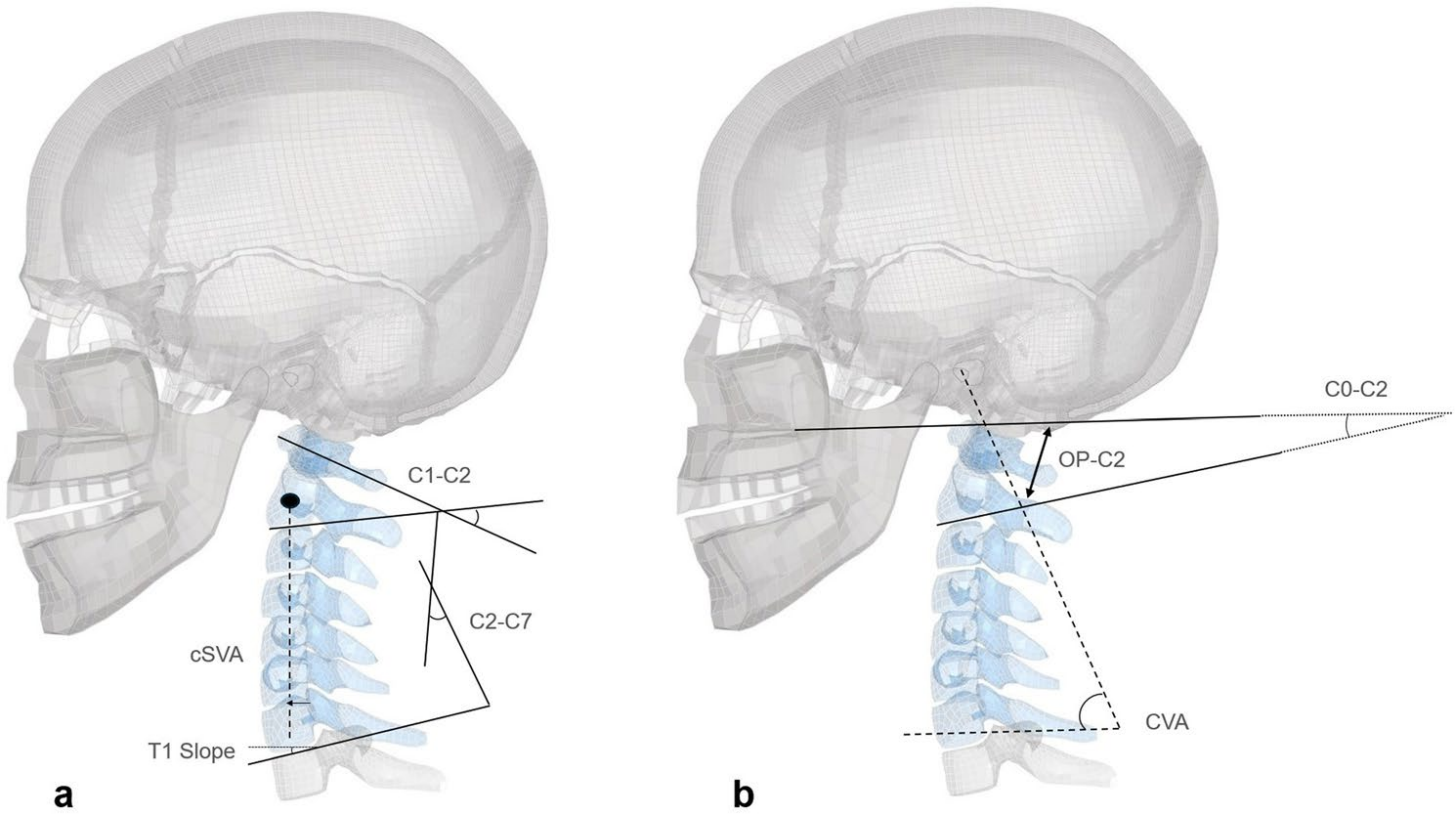
Radiographic representation of cervical and cranio-cervical sagittal balance parameters. **a** Subaxial and cervico-thoracic parameters, including, C1–C2 angle, C2–C7 lordosis, cervical sagittal vertical axis (cSVA), and T1 slope (**b**) Cranio-cervical parameters, including C0–C2 angle, OP–C2 distance, McGregor line, and craniovertebral angle (CVA), illustrating upper cervical orientation and head translation relative to the cervical spine

**Fig. 3:**
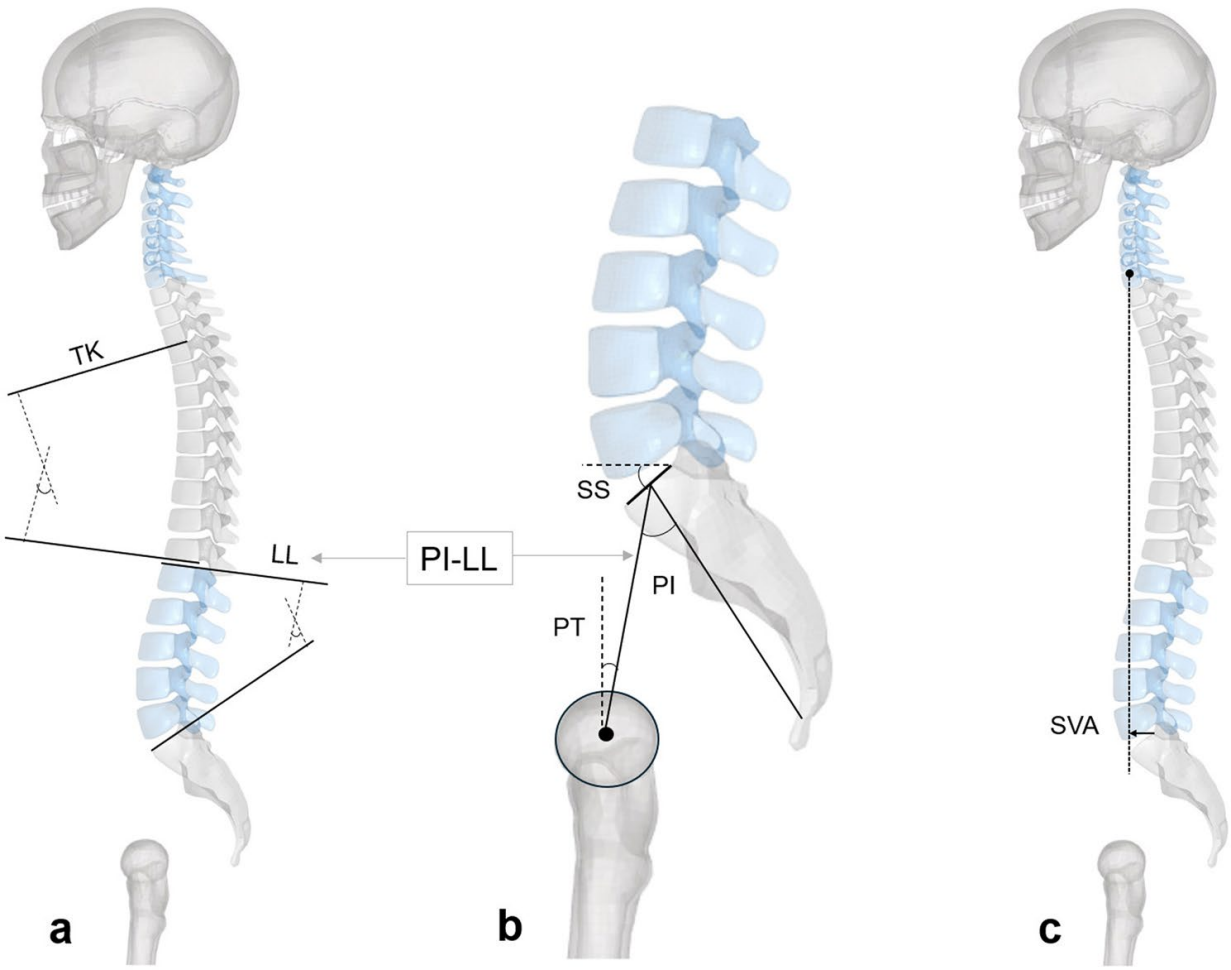
Radiographic representation of thoracic, lumbopelvic, and global sagittal balance parameters. **a** Thoracic kyphosis (TK) and lumbar lordosis (LL). **b** Spinopelvic parameters, including pelvic incidence (PI), pelvic tilt (PT), sacral slope (SS), and the pelvic incidence–lumbar lordosis (PI–LL) relationship. **c** Global sagittal vertical axis (SVA) illustrating overall sagittal alignment relative to the pelvis

Established radiographic definitions were used for anatomical landmarks of SB parameters (Table 1; Figs. 2 and 3). Each image was manually annotated and digitized, and two-dimensional landmark coordinates were recorded. All landmarking was performed by one primary rater across the full cohort (*n* = 201) using established radiographic definitions and a standardized protocol. Inter-rater reliability was assessed by a second independent evaluator with prior experience in radiographic sagittal balance assessment who repeated measurements on a subset of 40 radiographs, while intra-rater reliability was evaluated through repeat measurements performed by the primary evaluator on the same subset after a two-week interval while blinded to the initial results. Both evaluators were trained using the same landmark identification protocol before analysis. Agreement was quantified using intraclass correlation coefficients based on a two-way random-effects model for absolute agreement (ICC [1, 2]), and corresponding 95% confidence intervals were calculated. Formal blinding to FHP group allocation was not implemented, as sagittal alignment characteristics are inherently visible on lateral radiographs; nevertheless, group classification relied on predefined quantitative criteria.

**Table 1:** Definition and anatomical reference of sagittal balance parameters

Parameters	Normative Values	Anomical definition of the parameters	References
C0-C1 (°)	10.9 ± 6.7°	Angle between the McGregor line (posterior hard palate to opisthion) and the superior surface of the C1 ring	[26]
C0-C2 (°)	15.81 ± 7.15°	Angle between the McGregor line (posterior hard palate to opisthion) and the inferior endplate of C2	[16]
C1-C2 (°)	-35.5° to -26.5°	Outline of the C2, from anterior to posterior, and a line at the anterior point of the C1 ring	[16, 27, 28]
C2-C7 (°)	4.89 ± 12.84°	Outline between the C2 endplate and the lower C7 endplate	[16]
McGregor (°)	8.8-10.2°	Angle between the line connecting the posterior margin of the hard palate to the opisthion and the horizontal reference line	[29, 30]
OP-C2 (mm)	Decrease OP-C2	Distance between the occipital protuberance and the superior midpoint of the C2 spinous process, the distance in millimeters	[16, 48]
CVA (°)	Decrease CVA	The angle betwween the line from the external auditory meatus to the seventh cervical vertebra and a horizontal line through the seventh cervical vertebra	[16]
cSVA (mm)	Increased cSVA	The horizontal offset from the plumbline drop from C2 to the postero-superior corner of C7	[17]
T1 Slope (°)	26 ± 10°	The angle beween the horizontal and the caudal endplate of T1	[27]
TK (°)	40.6 ± 10°	The angle beween the caudal endplate of T12 and cranial endplate of T4	[31]
LL (°)	-57.4 ± 11.3°	The angle beween the upper endplate of L1 and sacral endplate	[31]
PI (°)	51° ± 10°	The angle beween a line from the center of femoral axis to the midpoint of the sacral plate and the perpendicular to the sacral plate	[27, 32]
PT (°)	13 ± 6°	A line from the centre of the femoral head axis to the midpoint of the sacral plate and the vertical	[27, 33, 34]
SS (°)	41 ± 8°	The angle between sacral endplate (S1) and the horizontal	[27, 33]
PI-LL (°)	Less than 10°	Defined as the difference between PI and LL. (PI=SS+PT)	[27, 35]
SVA (mm)	0.1±23mm	The horizontal offset from a plumbline dropped from C7 to the posterior-superior corner of S1	[27, 36]

Schematic anatomical representations of the sagittal balance parameters are provided in Figures 2 and 3

Angular and linear measurements were calculated based on standard geometric definitions for sagittal alignment. Angular parameters were derived from the relative orientation of landmark-defined vectors and were therefore independent of absolute image scale. Visualization and verification of landmark placement and measurement geometry were performed using the Matplotlib library. All computed parameters were compiled and exported into structured spreadsheets (Microsoft Excel format) for subsequent statistical analysis. Due to anatomical overlap, image field limitations, or insufficient visualization of specific landmarks on EOS images, not all sagittal parameters could be measured in every participant. As a result, the sample size varied slightly across individual radiographic measurements. Analyses were conducted using all available data for each parameter, and the corresponding sample size (n) is reported for each variable in the tables. No imputation was performed. To assess potential bias related to missing data, age and sex were compared between participants with and without available measurements for key sagittal balance parameters, and no clinically meaningful differences were observed.

### Ideal lumbar lordosis estimation

To provide an individualized reference for lumbar alignment relative to pelvic morphology and age, ideal lumbar lordosis was estimated for each subject using a PI- and age-adjusted predictive equation derived from asymptomatic adult populations [36]:

$$\:\mathrm{L}\mathrm{L}_\mathrm{ideal}=32.9+0.60\times\mathrm{PI}-0.23\times\mathrm{Age}$$

where LL and PI are expressed in degrees, and age in years. In this regression model, the intercept (32.9) represents the constant term of the equation, while the coefficients indicate that LL increases by approximately 0.60° per degree of PI and decreases by approximately 0.23° per year of age, as derived from asymptomatic adults using least-squares regression.

Deviation from ideal lumbar lordosis (ΔLL) was calculated as the difference between measured and predicted ideal lumbar lordosis, defined as:

$$\:\left(\triangle\mathrm{L}\mathrm{L}\:=\:\mathrm{L}\mathrm{L}_\mathrm{measured}\:-\:\mathrm{L}\mathrm{L}_\mathrm{ideal}\right.$$)

This deviation was used for subsequent group comparisons and categorical analyses. Participants were stratified as under-lordotic (ΔLL ≤ − 5°), within the ideal range (− 5° < ΔLL < + 5°), or over-lordotic (ΔLL ≥ + 5°). The ± 5° threshold was selected to exceed the minimum detectable change reported for lumbar Cobb measurements and to align with established clinical conventions indicating meaningful angular deviation beyond measurement variability [37, 38].

### Statistical analysis

Descriptive statistics were calculated for all SB parameters and are reported as mean ± standard deviation. Between-group comparisons between the FHP and non-FHP groups were performed using Welch’s t-test to account for unequal sample sizes and potential heterogeneity of variance. A two-sided p-value < 0.05 was considered statistically significant. Effect sizes were calculated using Cohen’s d to describe the magnitude of between-group differences independently of sample size. Cohen’s d values were interpreted using conventional thresholds as small (0.2), moderate (0.5), and large (0.8) effects.

To evaluate the potential influence of age differences between groups, age-adjusted linear regression analyses were performed. An initial model was applied to craniovertebral angle (CVA), and additional age-adjusted models were subsequently performed for sagittal alignment parameters demonstrating significant between-group differences (T1 slope, thoracic kyphosis [TK], and sagittal vertical axis [SVA]). In each model, group (FHP/non-FHP) and age were included as independent variables.

To explore relationships among SB parameters, an exploratory correlation analysis was conducted separately within the non-FHP and FHP groups. Associations were assessed using Spearman’s rank correlation coefficient (ρ), chosen for its robustness to non-normal distributions and outliers. Correlation matrices were generated for each group to describe group-specific patterns of association. For descriptive interpretation, correlation magnitudes were classified as weak (|ρ| < 0.30), moderate (|ρ| = 0.30–0.50), and strong (|ρ| > 0.50), in line with commonly used conventions in biomechanical and clinical research [39]. Accordingly, no correction for multiple comparisons was applied, and correlation results were interpreted descriptively based on the magnitude and structure of associations rather than on individual p-values. Because this involved multiple pairwise tests, we did not use correlation p-values for formal inference.

This exploratory analysis was intended to describe group-specific alignment patterns and to generate hypotheses regarding potential multi-level SB interactions rather than to infer causal relationships. Particular attention was given to associations observed in the FHP group that were absent or markedly weaker in the non-FHP group. To avoid overinterpretation, between group differences in correlation strength were interpreted qualitatively; formal testing for differences between correlations were not the primary aim. A large language model (ChatGPT, OpenAI) was used only to assist with English language editing and grammar correction.

## Results

### Demographic and clinical characteristics

**Fig. 4:**
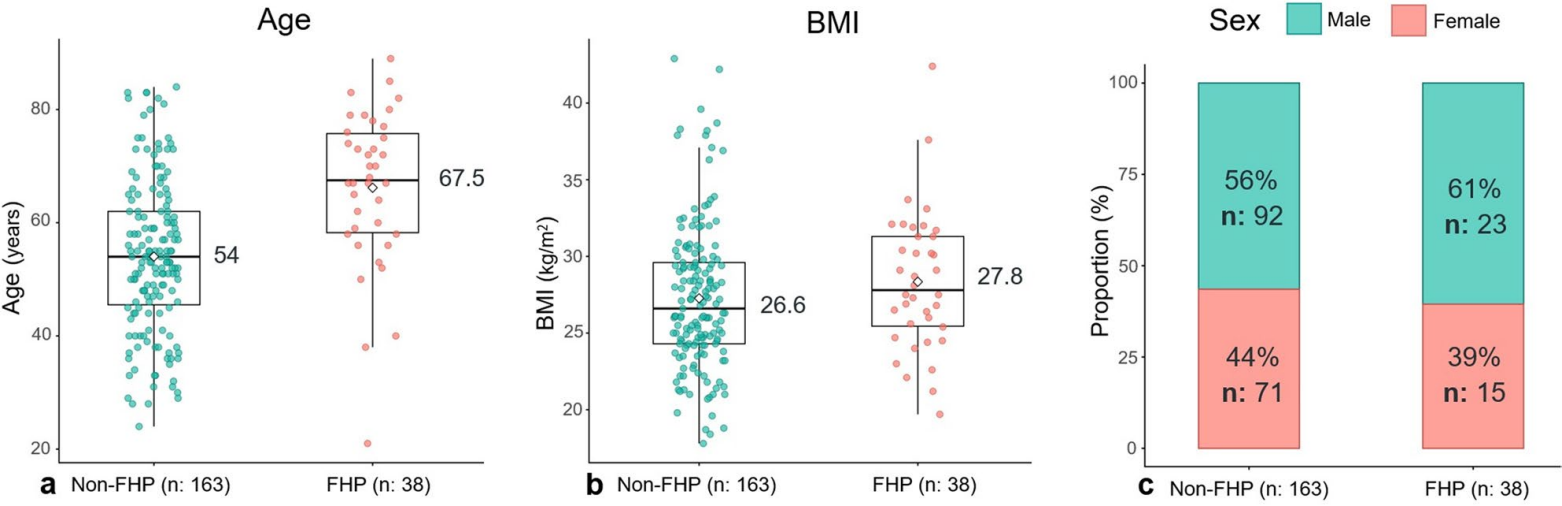
Demographic characteristics of the non-FHP and FHP groups (**a**) Age, (**b**) body mass index (BMI), and (**c**) sex distribution

A total of 201 individuals were included in the final analysis, with 163 classified as non-FHP and 38 as FHP based on CVA stratification. Demographic characteristics of both groups are summarized below (Table 2; Fig. 4).

**Table 2:** Demographic characteristics of the study cohort

Group	Non-FHP (*n*:163)	FHP (*n*:38)	*p*-value
Age (years) mean ± SD	54.0 ± 13.4	66.2 ± 14.0	< 0.001
BMI (kg/m²) mean ± SD	27.3 ± 4.7	28.3 ± 4.5	0.194
Male, n (%)	92 (56.4%)	23 (60.5%)	0.782

#### Age

Participants in the FHP group were older compared with those in the non-FHP group. The age distribution demonstrated a rightward shift in the FHP cohort, with higher median and upper quartile values relative to the non-FHP group (Fig. 4a). Age distributions partially overlapped between groups.

#### Age-adjusted analysis

In a linear regression model with CVA as the outcome and group status and age as predictors, individuals classified with FHP exhibited a CVA approximately 15.43° lower than the non-FHP group (*p* < 0.001). Age was significantly and negatively associated with CVA, with a decrease of 0.08° per year (*p* = 0.007). Overall, the model explained 57.3% of the variance in CVA (Table 3).

**Table 3:** Age-adjusted linear regression model for craniovertebral angle according to group

Parameter	Estimate (°)	*p*-value
Intercept non-FHP	65.30	< 0.001
FHP	–15.43	< 0.001
Age	–0.08	0.007
Multiple R²	0.573	—

Age-adjusted linear regression analyses are presented in (Table 4). FHP status remained significantly associated with increased T1 slope, TK, and SVA after adjustment for age. Age was significantly associated with T1 slope and SVA, but not with TK.

**Table 4:** Age-adjusted linear regression analyses of T1 slope, thoracic kyphosis (TK), and sagittal vertical axis (SVA) according to FHP status

Outcome	FHP Estimate	*p*-value	Age Estimate	*p*-value	*R*²
T1 slope(°)	7.92	< 0.001	0.14	0.009	0.200
TK (°)	5.91	0.014	0.09	0.159	0.059
SVA (mm)	17.15	0.007	0.74	< 0.001	0.172

FHP estimates represent the adjusted difference between the FHP and non-FHP groups after controlling for age. Non-FHP served as the reference group

#### Body mass index

Body mass index (BMI) distributions were broadly comparable between groups. The FHP group showed a slightly higher median BMI and greater dispersion; however, substantial overlap between groups was observed (Fig. 4b).

#### Sex distribution

Sex distribution was similar between groups. In the non-FHP group, males accounted for 56% of participants, while females represented 44%. In the FHP group, males comprised 61% of the cohort and females 39% (Fig. 4c). No substantial sex imbalance was observed between groups (*p* = 0.782).

### Inter- and intra-rater reliability

Inter- and intra-rater reliability of sagittal balance measurements were generally high (Table 5). Excellent agreement (ICC ≥ 0.90) was observed for the majority of linear and spinopelvic parameters, including CVA, cSVA, SVA, and pelvic measures. Cervico-thoracic parameters such as T1 slope and thoracic kyphosis also demonstrated excellent reproducibility. Moderate-to-good agreement was observed for selected cranio-cervical angles, including C0–C1 and C0–C2, reflecting greater landmark sensitivity in the upper cervical region. This region is characterized by complex craniovertebral anatomy and closely spaced anatomical landmarks, where small differences in landmark identification may have a proportionally greater effect on angular measurements than in larger spinopelvic or global alignment parameters.

**Table 5:** Inter- and intra-rater reliability of sagittal balance parameters measured on standing EOS radiographs using ICC (2,1)

	Inter-rater				Intra-rater			
Parameter	n	ICC (2,1)	95% CI	Interpretation	n	ICC (2,1)	95% CI	Interpretation
C0–C1 (°)	31	0.6229	0.32–0.81	Moderate	30	0.789	0.57–0.90	Good
C0–C2 (°)	31	0.7809	0.49–0.90	Good	30	0.7172	0.40–0.87	Moderate
C1-C2 (°)	40	0.8817	0.79–0.94	Good	40	0.7898	0.63–0.88	Good
C2-C7 (°)	36	0.8104	0.66–0.90	Good	34	0.6863	0.46–0.83	Moderate
McGregor (°)	31	0.7788	0.59–0.89	Good	30	0.7403	0.53–0.87	Moderate
OP–C2 (mm)	39	0.9789	0.96–0.99	Excellent	39	0.9694	0.91–0.99	Excellent
CVA (°)	40	0.9991	1.00–1.00	Excellent	40	0.9973	0.99–1.00	Excellent
cSVA (mm)	38	0.9988	1.00–1.00	Excellent	36	0.9981	1.00–1.00	Excellent
T1 Slope (°)	28	0.94	0.87–0.97	Excellent	27	0.922	0.84–0.96	Excellent
TK (°)	39	0.9749	0.95–0.99	Excellent	39	0.9254	0.86–0.96	Excellent
LL (°)	40	0.9584	0.92–0.98	Excellent	40	0.9708	0.95–0.98	Excellent
PI (°)	40	0.9708	0.95–0.98	Excellent	40	0.9728	0.95–0.99	Excellent
PT (°)	40	0.9888	0.98–0.99	Excellent	40	0.9886	0.98–0.99	Excellent
SS (°)	40	0.9467	0.90–0.97	Excellent	40	0.9602	0.93–0.98	Excellent
PI–LL (°)	40	0.9864	0.97–0.99	Excellent	40	0.9874	0.98–0.99	Excellent
SVA (mm)	38	0.9993	1.00–1.00	Excellent	36	0.9989	1.00–1.00	Excellent

### Group-wise comparison of sagittal balance parameters

SB parameters differed between the FHP and non-FHP groups across multiple spinal regions. At the upper cervical level, the FHP group demonstrated significantly greater C0–C2 and C1–C2 angles, together with a higher McGregor angle and a shorter OP–C2 distance. CVA was significantly reduced in the FHP group. In contrast, C0–C1 did not differ significantly between groups (Table 6).

**Table 6:** Comparison of sagittal balance parameters between non-FHP and FHP groups

Parameters	Non-FHP		FHP			
	*n*	Mean ± SD	*n*	Mean ± SD	*p*-value	Cohen’s d
C0–C1 (°)	152	14 ± 7.3	27	11 ± 7.6	0.063	-0.41
C0–C2 (°)	152	18.1 ± 9.5	27	27.7 ± 11.7	**< 0.001**	0.97
C1-C2 (°)	163	31.8 ± 8.2	38	36.5 ± 8.5	**0.003**	0.57
C2-C7 (°)	161	14.1 ± 10.3	35	12.9 ± 8.1	0.459	-0.12
McGregor (°)	151	5.6 ± 4.0	27	7.8 ± 5.2	**0.040**	0.52
OP–C2 (mm)	160	28 ± 5	37	25 ± 7	**0.006**	-0.55
CVA (°)	163	61.0 ± 5.9	38	44.6 ± 5.2	**< 0.001**	-2.84
cSVA (mm)	159	22 ± 10	34	43 ± 12	**< 0.001**	2.02
T1 Slope (°)	117	31.7 ± 8.9	29	41.5 ± 9.1	**< 0.001**	1.10
TK (°)	160	42.4 ± 11.6	36	49.5 ± 14.4	**0.008**	0.58
LL (°)	163	49.8 ± 13.4	38	48.6 ± 16.9	0.691	-0.09
PI (°)	162	52.4 ± 11.8	38	54.0 ± 13.5	0.499	0.13
PT (°)	162	13.7 ± 7.6	38	16.5 ± 9.2	0.090	0.35
SS (°)	163	39.1 ± 9.8	38	38.0 ± 13.3	0.662	-0.10
PI–LL (°)	162	2.5 ± 11.6	38	5.3 ± 13.2	0.225	0.23
SVA (mm)	159	37 ± 32	34	64 ± 37	**< 0.001**	0.82

Bold **p**-values indicate statistically significant differences (**P** < 0.05)

At the sub-axial cervical level, C2–C7 lordosis did not differ significantly between groups, whereas cervical cSVA was significantly greater in the FHP group. At the thoracic level, the FHP group exhibited significantly higher T1 slope and greater TK. Cohen’s d values indicated large effect sizes for CVA, cSVA, and T1 slope, moderate effects for upper cervical and thoracic parameters, and small effects for subaxial cervical and lumbopelvic measures (Table 6).

At the lumbo-pelvic level, LL and spinopelvic parameters (PI, PT, SS and PI–LL mismatch) did not differ significantly between groups. Regarding global alignment, SVA was significantly larger in the FHP group (Table 6).

### Correlation analysis of sagittal balance parameters

#### Overall correlation structure

**Fig. 5:**
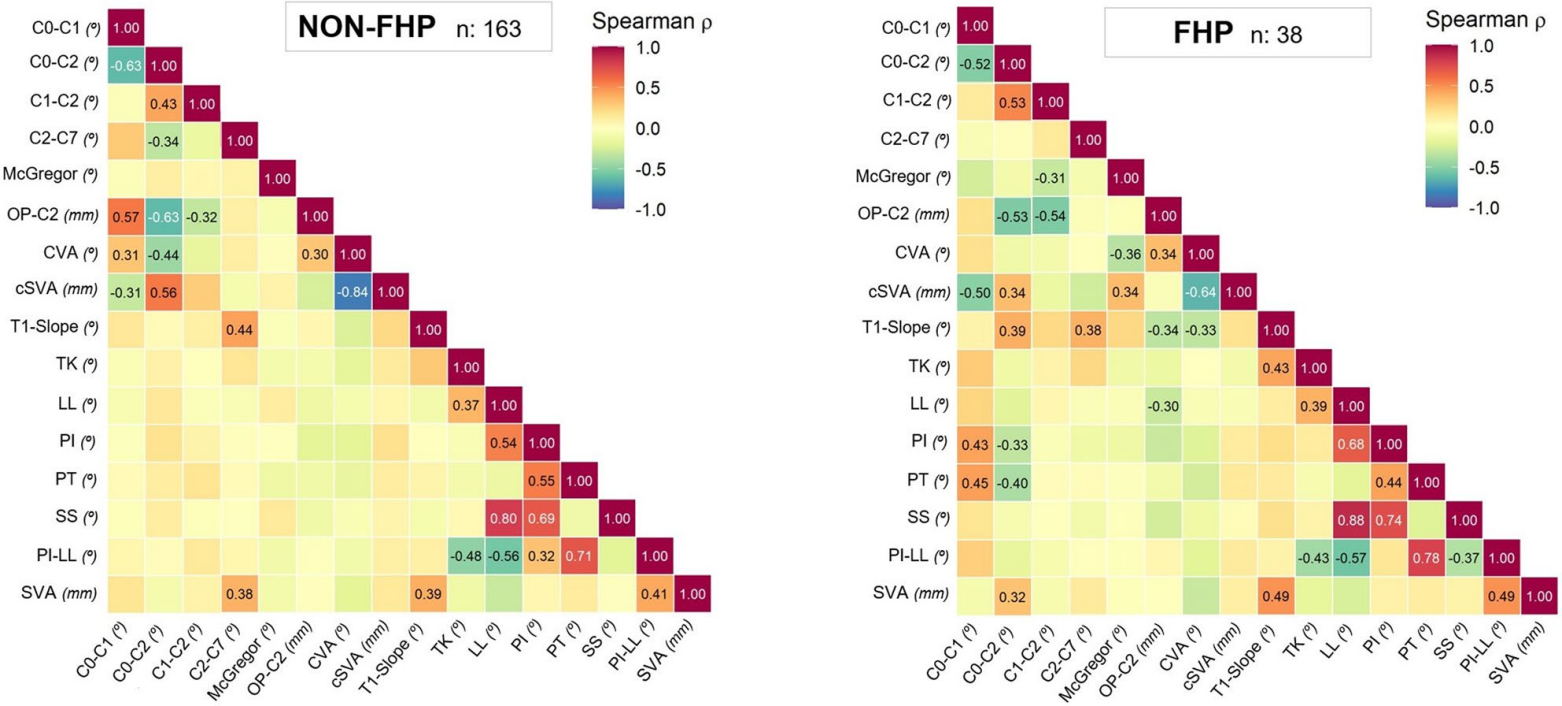
Spearman correlation matrices of sagittal balance parameters in (**a**) the non-FHP group and (**b**) the FHP group. Colours indicate the strength and direction of associations (Spearman ρ). For clarity, only correlations with an absolute magnitude of |ρ| ≥ 0.30 are displayed

Correlation matrices for each group are presented in Fig. 5. In the non-FHP group, correlations were predominantly localized within each anatomical region, with stronger associations observed among cervical parameters and among lumbopelvic measures (e.g., CVA vs. cSVA: ρ = −0.84; PI vs. SS: ρ = 0.69). Cross-regional correlations linking cervical alignment to thoracic, lumbopelvic, or global parameters were limited in magnitude and frequency.

In contrast, the FHP group demonstrated a broader correlation structure, characterized by a greater number of moderate associations extending beyond the cervical region. Several cervical and cranio-cervical parameters showed correlations with thoracic orientation and global alignment measures that were not observed in the non-FHP group.

#### Cervical and cervico-thoracic associations

Across both groups, lower cervical lordosis (C2–C7) maintained a consistent association with T1 slope, reflecting stable cervico-thoracic coupling. Upper cervical parameters, particularly C0-C1, C0–C2 and C1–C2, showed limited associations in the non-FHP group, largely confined to local cranio-cervical measures.

In the FHP group, upper cervical alignment parameters exhibited broader associations, including correlations with T1 slope, (cSVA), pelvic parameters and head orientation metrics such as the McGregor angle and CVA. These associations were absent in the non-FHP cohort (e.g., C0-C2 vs. SVA: ρ = 0.32; C0-C2 vs. PT: 0.45).

#### Group-specific correlation patterns in FHP

**Fig. 6:**
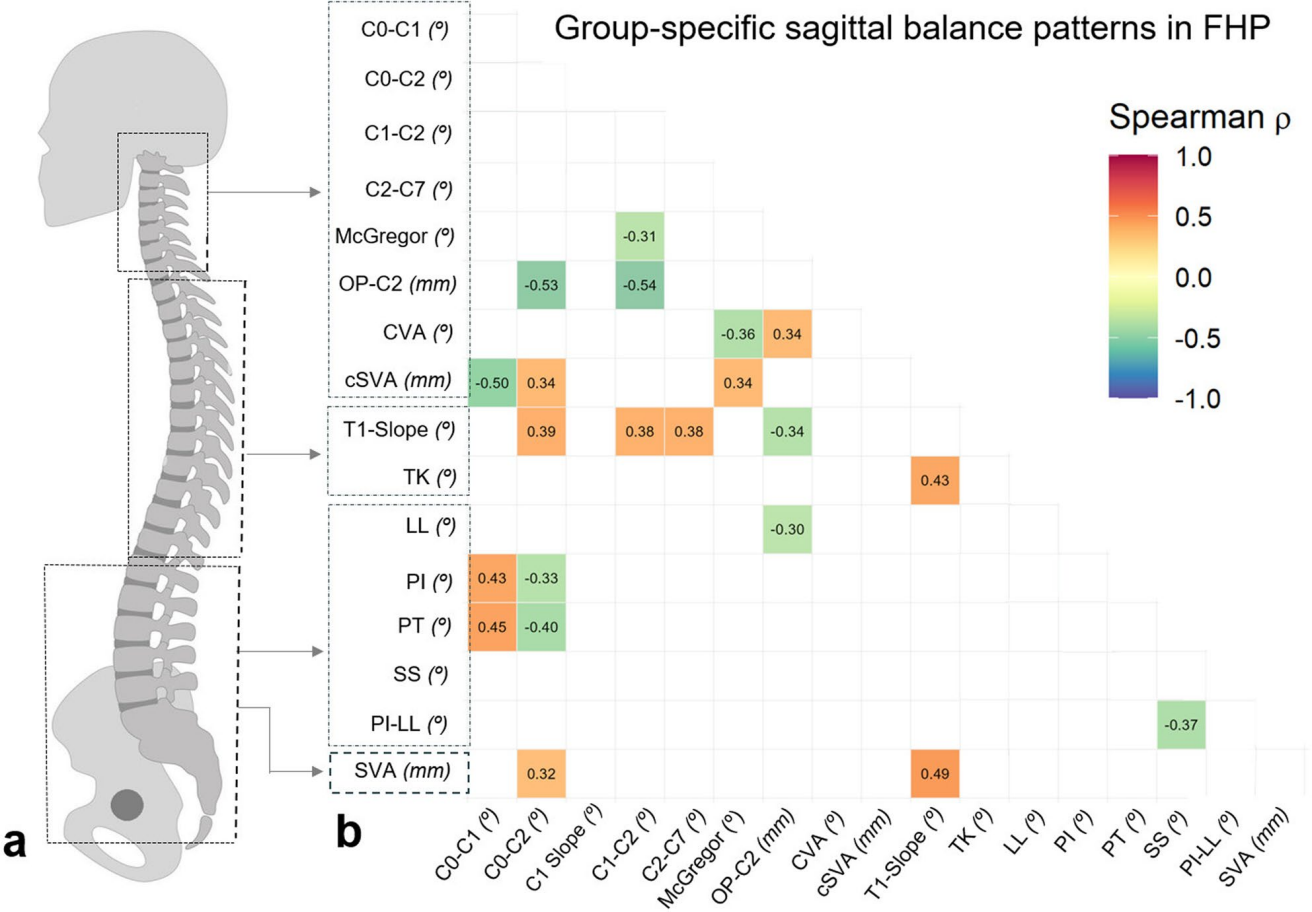
**a** Schematic representation of spinal regions included in the analysis. Cervical, thoracic, lumbopelvic, and global parameters are organized by anatomical region to highlight cross-regional relationships. This figure was included to provide a simplified visualization of the principal cross-regional associations observed in the FHP cohort. **b** Spearman correlation matrix of sagittal balance parameters in the FHP group. Colour indicates the strength and direction of associations (Spearman ρ). Only correlations with an absolute magnitude of |ρ| ≥ 0.30 are displayed

To facilitate interpretation of the broader correlation matrices, Fig. 6 highlights the principal FHP-specific associations identified in the exploratory analysis. A subset of correlations was observed exclusively in the FHP group (Fig. 6). These included associations between upper cervical parameters (C0–C1, C0–C2) and lumbopelvic measures such as PT and PI. C0-C2, in particular, was associated with SVA. In addition, OP–C2 distance showed correlations with T1 slope and LL (e.g., OP–C2 vs. T1 slope: ρ = −0.34; OP–C2 vs. LL: ρ = −0.30).Additionally, thoracic orientation parameters, particularly T1 slope and TK (T1 slope vs. TK: ρ = 0.43) demonstrated stronger associations with global SVA in the FHP group compared with the non-FHP group. Lumbopelvic parameters retained their expected internal correlations in both groups, with slightly increased correlation strength in the FHP cohort. Overall, the FHP group displayed a broader network of associations connecting cranio-cervical alignment with thoracic and global SB measures, whereas the non-FHP group exhibited a more regionally constrained correlation pattern. Given the exploratory nature of the analysis and the absence of adjustment for multiple comparisons, these associations should be interpreted as descriptive patterns intended to generate hypotheses rather than as evidence of definitive mechanistic relationships.

### Ideal lumbar lordosis analysis

**Fig. 7:**
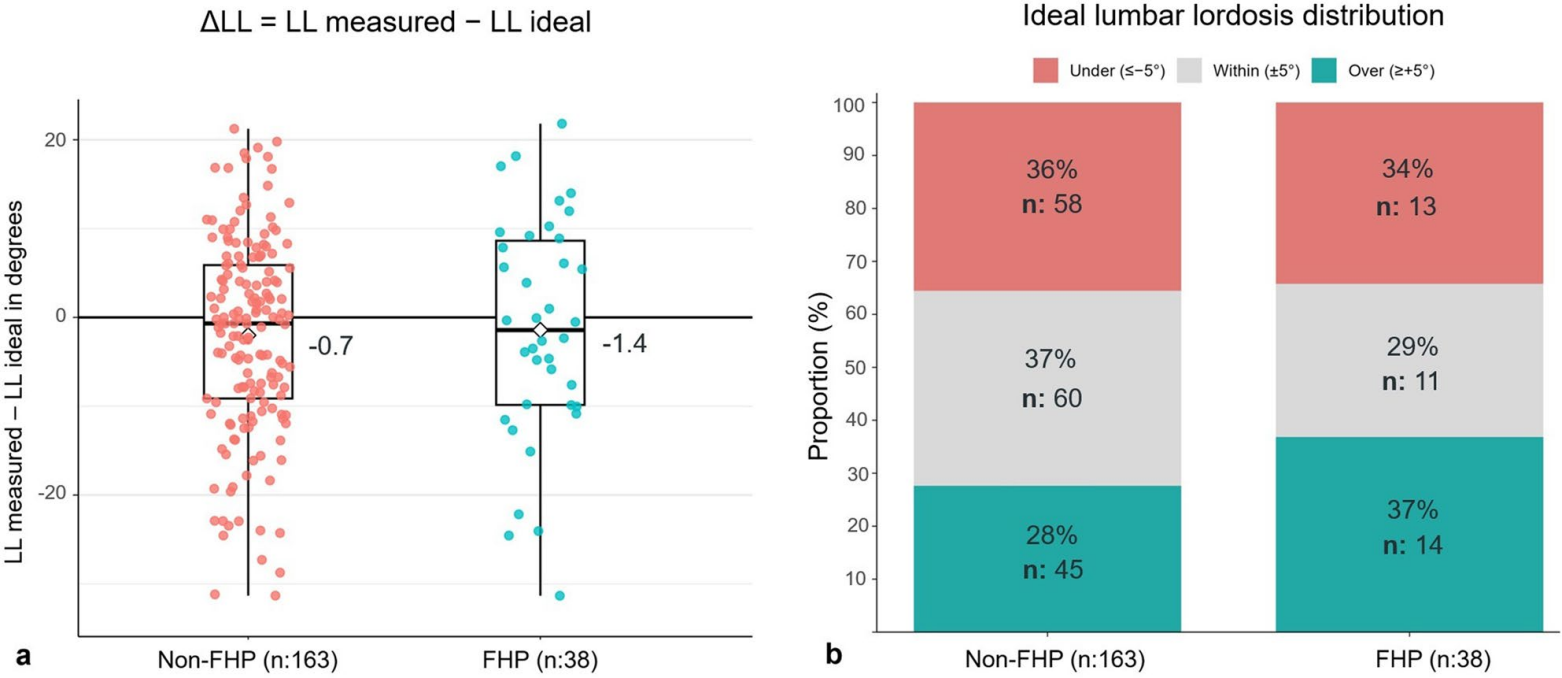
Ideal Lumbar Lordosis (**a**) the deviation from the ideal lordosis and (**b**) the distribution of LL

The distribution of ΔLL values showed substantial overlap between the non-FHP and FHP groups (Fig. 7). Median ΔLL values in both groups were close to zero, indicating comparable average alignment relative to $\:\mathrm{L}\mathrm{L}_\mathrm{ideal}$. Despite similar central tendencies, the FHP group exhibited greater variability, with a wider distribution of both negative and positive deviations from $\:\mathrm{L}\mathrm{L}_\mathrm{ideal}$.

Participants were further categorized according to predefined clinical thresholds as under-lordotic (ΔLL ≤ − 5°), within the ideal range (− 5° < ΔLL < + 5°), or over-lordotic (ΔLL ≥ + 5°). In the FHP group, 34% of individuals were classified as under-lordotic, 29% within the ideal range, and 37% as over-lordotic. In the non-FHP group, 36% were under-lordotic, 37% within the ideal range, and 28% were over-lordotic (Fig. 7).

## Discussion

### Demographic and clinical characteristics

This study investigated whether FHP is associated with alterations in global sagittal alignment. Using standing EOS imaging, it was found that individuals with FHP exhibit distinct upper cervical and cervico-thoracic alignment features, extending into global sagittal organization. These findings suggest that FHP is part of a more extensive postural alignment pattern rather than representing an isolated cervical phenomenon.

The demographic characteristics of the participants demonstrated that the FHP group were older than those in the non-FHP group. Given the established influence of age on sagittal alignment [40–42], the performed age-adjusted linear regression analysis showed that group status remained independently associated with CVA after adjustment for age. These findings suggest that the observed CVA differences are not solely explained by age-related changes. The influence of age on whole-spine sagittal alignment has been widely reported, with progressive increases in thoracic kyphosis, T1 slope, and sagittal vertical axis occurring across the lifespan. Previous studies have demonstrated that age-related changes contribute substantially to thoracic and global alignment parameters, particularly SVA [40]. Despite these established age-related trends, additional age-adjusted analyses demonstrated that FHP status remained independently associated with increased T1 slope, TK, and SVA. These findings suggest that the observed between-group differences cannot be attributed solely to age-related sagittal alignment changes and support an independent association between FHP and alterations in cervicothoracic and global alignment. Nevertheless, the marked age difference between groups should be considered when interpreting these findings. Given the established influence of ageing on thoracic kyphosis, sagittal vertical axis, and overall sagittal alignment, residual age-related effects cannot be completely excluded despite statistical adjustment. BMI did not differ substantially between groups, suggesting that sagittal alignment differences were unlikely to be primarily attributable to body habitus. However, it should be noted that associations between BMI, spinal alignment [43] and head and neck posture [44], have been reported in previous studies. Sex distribution was broadly comparable between groups, with a slightly higher proportion of male participants in the FHP cohort, consistent with previously reported sex-related patterns in FHP [45]. Measurement reliability was high overall, as confirmed by inter- and intra-rater intraclass correlation coefficients (ICC), supporting the robustness of the sagittal alignment analyses, although slightly lower reproducibility was observed in selected upper cervical parameters.

### Group-wise comparison of sagittal balance parameters

Sixteen SB parameters were compared between the non-FHP and FHP groups. At the upper cervical level, the FHP group showed significantly larger C1–C2 and C0–C2 angles and a higher McGregor angle, consistent with a cranio-cervical extension pattern [46]. Although C2–C7 lordosis showed a non-significant decreasing trend in the FHP group, the direction of this change aligns with the inverse upper–lower cervical curvature pattern described in previous studies [16, 17].

OP–C2 was significantly shorter in FHP, reflecting suboccipital involvement described in previous work studies [16, 17, 47, 48]. These findings are consistent with previously reported associations between head orientation and whole-spine sagittal alignment [18, 22, 49]. CVA was significantly lower in the FHP group, reflecting a more anterior head position consistent with reported moderate-to-severe FHP ranges [50], while non-FHP values fell within normative limits [51].

In the FHP group, cSVA, T1 slope, TK, and SVA were all significantly higher. This pattern aligns with previously described associations between cSVA and T1 slope [18, 52, 53], as well as reported relationships linking higher T1 slope with greater TK [54] and with increased SVA [55]. Notably, the associations between FHP status and T1 slope, TK, and SVA remained significant after adjustment for age, further supporting the involvement of the cervico-thoracic region in the alignment patterns observed in FHP.

Pelvic and LL parameters did not differ significantly between groups, although PI–LL mismatch values were higher in the FHP group. This pattern suggests that global sagittal alignment changes may occur in FHP in the absence of fixed lumbo-pelvic compensation, a phenomenon previously described within whole-spine sagittal balance frameworks [10, 56, 57]. As all participants were referred for evaluation of low back pain, it cannot be excluded that pain-related adaptations influenced certain aspects of lumbo-pelvic and global alignment. Nevertheless, the absence of significant between-group differences in most pelvic and lumbar parameters suggests that the observed FHP-related alignment characteristics were not accompanied by major alterations in lumbo-pelvic alignment within this clinical population.

### Sagittal balance correlations and coordinated alignment

Correlations were analysed separately in both groups to explore associations unique or markedly different in the FHP group as part of an exploratory analysis aimed at identifying alignment metrics most strongly involved in FHP-related sagittal organization. This approach was intended to complement the predefined group comparisons rather than to test additional confirmatory hypotheses. Given the unequal group sizes, particularly the smaller FHP cohort, the resulting association patterns should be interpreted cautiously, as some correlations may be sensitive to sampling variability. At the cervical level in FHP group, McGregor’s line was correlated with both cSVA and CVA, highlighting the relevance of the gaze horizontalization within global FHP metrics [46]. In contrast, in the non-FHP group, upper cervical parameters C0-C2 were correlated with the sub axial alignment C2–C7 [18] and with CVA. These association were absent in the FHP group, along with the correlation between C2–C7 and SVA observed in non-FHP individuals, despite C2–C7 being commonly considered representative of global cervical alignment [18, 58] and widely used in SB studies [59, 60]. Notably, m between C0–C2 and CVA in the FHP group suggests that upper cervical adaptations may not be fully captured by CVA alone, despite its widespread use for FHP diagnosis and clinical classification [22, 24, 61]. In the present study, CVA was used as a pragmatic stratification criterion rather than as a comprehensive descriptor of sagittal alignment. The observed dissociation between CVA and several regional alignment relationships in the FHP cohort suggests that, although useful for cohort classification, CVA alone may not fully characterize the underlying organization of whole-spine sagittal alignment. Together, these findings suggest that alignment relationships derived from non-FHP populations may not fully apply to the alignment patterns observed in FHP. Additionally, C0-C1 and C0-C2 correlated with pelvic parameters such as PI and PT, exclusively in FHP group. In the same cohort, C0-C2 was also associated with T1 slope and SVA. OP-C2 correlated with CVA in both groups, however, only in FHP group OP-C2 was associated with T1 slope and LL, representing the only upper cervical parameter directly related to LL. Given that OP–C2 is not yet a standardized SB metric, this finding should be interpreted with caution [48].

Multiple cervical parameters demonstrated broader associations with T1 slope only in FHP group, consistent with previous descriptions of T1 slope as an important cervico-thoracic sagittal alignment [18]. Nevertheless, C2–C7 lordosis showed the expected association with T1 slope in both groups, reflecting preserved cervico-thoracic coupling [17, 59]. Notably, T1 slope was additionally correlated with TK and SVA only in FHP group, consistent with biomechanical descriptions of T1 slope as a pivot in global sagittal alignment [62].

Globally, TK maintained its expected relationships with LL and PI–LL [35, 57], and LL showed preserved correlations with pelvic parameters [63] in both groups. This consistency may indicate that lumbo-pelvic alignment remains relatively preserved, with sagittal alterations in FHP appearing more prominent above the lumbar spine. However, these observations should be interpreted within the context of an exploratory correlation analysis. Several associations were observed exclusively in the FHP group; however, formal statistical comparisons between correlation coefficients were not performed. Consequently, these patterns should be regarded as descriptive observations that may help guide future investigations rather than as evidence of distinct biomechanical mechanisms.

### Ideal lumbar lordosis analysis

Analysis of the ideal lumbar lordosis showed no systematic deviation from predicted alignment in the FHP group compared with the non-FHP group, with mean ΔLL values close to zero in both cohorts, indicating preserved average lumbar alignment [21, 57]. Deviations from predicted lumbar alignment are typically reported in the presence of fixed structural spinal deformity, which was not represented in the present cohort [37, 59]. Although central tendencies were similar, the FHP group exhibited greater variability, with a wider distribution of under- and over-lordotic profiles, suggesting heterogeneous, potentially functional adaptations rather than a uniform shift. These observations should be interpreted as descriptive and hypothesis-generating rather than causal.

### Clinical relevance

The present findings offer clinically relevant considerations for interpreting sagittal alignment in individuals with FHP. Commonly used FHP metrics such as CVA and cSVA remain helpful descriptors of overall head–trunk positioning. However, their presence in both FHP and non-FHP individuals suggests that, when used alone, they may provide limited insight into regional cervical or whole-spine alignment patterns. In this context, the observed dissociation between CVA and C0-C2 behaviour in the FHP cohort indicates that global head translation measures may not fully capture adaptations occurring at the craniovertebral junction.

The findings suggest that alignment changes associated with FHP may be more evident at the upper cervical (C0-C1 and C0-C2) and cervico-thoracic levels (T1 slope and TK) and with the global SVA. In contrast, subaxial cervical C2-C7 and lumbo-pelvic alignment may appear relatively preserved. Therefore, although seemingly “normal”, these metrics should be interpreted with caution and not automatically considered indicative of overall sagittal neutrality in patients with FHP.

Taken together, these findings suggest that clinical evaluation of FHP should not rely solely on subaxial cervical or lumbo-pelvic parameters. Previous studies have reported associations between FHP and lumbopelvic sagittal alignment in individuals with chronic low back pain [22]. Although low back pain may influence standing posture, the most consistent differences observed in the present study involved upper cervical, cervico-thoracic, and global alignment parameters. In contrast, most lumbo-pelvic parameters remained relatively preserved. Incorporating assessment of the upper cervical region and cervico-thoracic alignment may provide a more complete understanding of sagittal alignment patterns in patients with FHP. Although symptom measures were unavailable in the present cohort, these findings may help guide radiographic assessment by identifying alignment regions that warrant consideration beyond conventional FHP metrics. It should also be recognized that the present groups were defined using a CVA-based threshold. Given the absence of a universally accepted diagnostic criterion for FHP, the findings may be interpreted as comparisons between lower-CVA and higher-CVA postural phenotypes rather than definitive diagnostic categories. Nevertheless, the observed alignment differences suggest that CVA-based stratification captures broader sagittal alignment characteristics extending beyond a single angular measurement.

### Limitations of the study

The findings should be interpreted in the context of the study design and population. Although age-adjusted analyses supported an independent association between group status and CVA, the older age of the FHP cohort should be considered when interpreting whole-spine sagittal alignment patterns, as age-related changes may have influenced some observed between-group differences. The cohort consisted of individuals referred for evaluation of low back pain; therefore, extrapolation to asymptomatic populations should be made cautiously, and pain-related postural adaptations cannot be fully excluded despite standardized EOS acquisition and measurement protocols. Future studies including asymptomatic populations may help determine the extent to which the observed alignment patterns are specific to FHP independent of the clinical characteristics of the present cohort. Clinical outcome measures, such as pain intensity, functional disability, and quality-of-life assessments, were not available in the retrospective dataset. Therefore, the relationship between the observed alignment patterns and patient symptom burden could not be evaluated. In addition, FHP was classified using a CVA-based threshold, acknowledging that head posture represents a continuous spectrum rather than a strictly dichotomous condition. Consequently, the groups may be more appropriately interpreted as lower-CVA and higher-CVA postural phenotypes rather than definitive diagnostic categories. The cross-sectional nature of the study does not permit determination of temporal or causal relationships between spinal regions. Therefore, it cannot be established whether the observed alignment patterns originate in the cervical spine and extend caudally or arise through alternative regional interactions. Finally, the correlation analyses were exploratory and not adjusted for multiple comparisons; accordingly, the observed relationships should be considered descriptive and hypothesis-generating rather than confirmatory or causal.

## Conclusion

Forward head posture was associated with a distinct head-to-pelvis sagittal alignment pattern on standing EOS images. The main differences involved the upper cervical spine and the cervico-thoracic junction, particularly T1 slope, while lumbar lordosis and spinopelvic morphology were largely preserved. In contrast, classical subaxial cervical metrics (C2–C7) appeared less informative in this context. These findings support consideration of upper cervical and cervico-thoracic parameters in the assessment of FHP, while the observed inter-regional relationships should be further explored in future studies.

## Data Availability

The datasets used and/or analysed during the current study are not publicly available due to institutional restrictions but are available from the corresponding author on reasonable request.

## Abbreviations

Abbreviation: Definition
BMI: Body mass index
CVA: Craniovertebral angle
cSVA: Cervical sagittal vertical axis
DICOM: Digital Imaging and Communications in Medicine
EOS: EOS imaging system
FHP: Forward head posture
ICC: Intraclass correlation coefficient
ICC (2,1): Intraclass correlation coefficient based on a two-way random-effects model for absolute agreement
LBP: Low back pain
LL: Lumbar lordosis
OP–C2: Occipital protuberance to C2 distance
PI: Pelvic incidence
PI–LL: Pelvic incidence–lumbar lordosis mismatch
PT: Pelvic tilt
SB: Sagittal balance
SD: Standard deviation
SS: Sacral slope
SVA: Sagittal vertical axis
TK: Thoracic kyphosis
ΔLL: Deviation from ideal lumbar lordosis
C0–C1: Occiput to atlas angle
C0–C2: Occiput to axis angle
C1–C2: Atlas to axis angle
C2–C7: Subaxial cervical lordosis angle
